# Sequence- and Structure-Based Immunoreactive Epitope Discovery for *Burkholderia pseudomallei* Flagellin

**DOI:** 10.1371/journal.pntd.0003917

**Published:** 2015-07-29

**Authors:** Arnone Nithichanon, Darawan Rinchai, Alessandro Gori, Patricia Lassaux, Claudio Peri, Oscar Conchillio-Solé, Mario Ferrer-Navarro, Louise J. Gourlay, Marco Nardini, Jordi Vila, Xavier Daura, Giorgio Colombo, Martino Bolognesi, Ganjana Lertmemonkolchai

**Affiliations:** 1 The Centre for Research and Development of Medical Diagnostic Laboratories, Faculty of Associated Medical Sciences, Khon Kaen University, Khon Kaen, Thailand; 2 Istituto di Chimica del Riconoscimento Molecolare, Consiglio Nazionale delle Ricerche, Milan, Italy; 3 Department of Biosciences, CIMAINA and CNR Institute of Biophysics, University of Milan, Milan, Italy; 4 Institute of Biotechnology and Biomedicine, Universitat Autònoma de Barcelona, Bellaterra, Spain; 5 Department of Clinical Microbiology, Hospital Clinic, School of Medicine, University of Barcelona, Barcelona, Spain; 6 Catalan Institution for Research and Advanced Studies, Barcelona, Spain; Charles Darwin University, AUSTRALIA

## Abstract

*Burkholderia pseudomallei* is a Gram-negative bacterium responsible for melioidosis, a serious and often fatal infectious disease that is poorly controlled by existing treatments. Due to its inherent resistance to the major antibiotic classes and its facultative intracellular pathogenicity, an effective vaccine would be extremely desirable, along with appropriate prevention and therapeutic management. One of the main subunit vaccine candidates is flagellin of *Burkholderia pseudomallei* (FliC_Bp_). Here, we present the high resolution crystal structure of FliC_Bp_ and report the synthesis and characterization of three peptides predicted to be both B and T cell FliC_Bp_ epitopes, by both structure-based *in silico* methods, and sequence-based epitope prediction tools. All three epitopes were shown to be immunoreactive against human IgG antibodies and to elicit cytokine production from human peripheral blood mononuclear cells. Furthermore, two of the peptides (F51-69 and F270-288) were found to be dominant immunoreactive epitopes, and their antibodies enhanced the bactericidal activities of purified human neutrophils. The epitopes derived from this study may represent potential melioidosis vaccine components.

## Author Summary

Melioidosis is an infectious disease caused by *Burkolderia pseudomallei* that poses a major public health problem in Southeast Asia and northern Australia. This bacterium is difficult to treat due to its intrinsic resistance to antibiotics, poor diagnosis, and the lack of a licensed vaccine. Vaccine safety is a prime concern, therefore recombinant protein subunit and/or peptide vaccine components, may represent safer alternatives. In this context, we targeted one of the main subunit vaccine candidates tested to date, flagellin from *B*. *pseudomallei* (FliC_Bp_) that comprises the flagellar filament that mediates bacterial motility. Based on the knowledge that activation of both cell-mediated and antibody-mediated responses must be addressed in a melioidosis vaccine, we identified B and T cell immunoreactive peptides from FliC_Bp_, using both sequence-based and structure-based computational prediction programs, for further *in vitro* immunological testing. Our data confirm the accuracy of sequence-based epitope prediction tools, and two structure-based methods applied to the FliC_Bp_ crystal structure (here-described), in predicting both T- and B-cell epitopes. Moreover, we identified two epitope peptides with significant joint T-cell and B-cell activities for further development as melioidosis vaccine components.

## Introduction

*Burkholderia pseudomallei*, a pathogenic Gram-negative bacterium present in soil and water, is responsible for melioidosis, an often fatal infectious disease that is most frequently reported in tropical regions of the world, especially in Thailand and northern Australia, where the disease is endemic [1]. Diagnosis and treatment of melioidosis are far from adequate, as symptoms lack a specific signature necessary for rapid diagnosis, and the bacterium is inherently resistant to many commercially available classes of antibiotics. In addition, due to the intrinsically polymorphic nature of the pathogen, infections can occur in acute and chronic forms, with a plethora of different clinical manifestations [2]. Over the last few years, the study of melioidosis has become increasingly relevant, not only as a public health concern, but also due to bioterrorism implications, since *B*. *pseudomallei* is classified as a category B infective agent.

To date, no melioidosis vaccine is available, and no vaccine candidates are close to licensing [3,4]. Furthermore, the use of attenuated forms of *B*. *pseudomallei* presents significant safety issues due to its capacity to remain dormant and cause infections years later, therefore it is clear that alternative approaches must be sought.

A subunit vaccine approach, whereby only the microbial components that produce an appropriate immune response are administered, offers a means to significantly improve vaccine safety [5]. One safer alternative is a peptide-based vaccine approach, since peptides are easily and inexpensively produced [6]. Although subunit vaccines present many desirable qualities, their ability to stimulate a potent immune responses is much weaker than traditional whole-cell preparations. This may explain why no effective vaccine candidates against *B*. *pseudomallei* infection have emerged, despite several attempts using different approaches [7].

According to previous studies, protection against *B*. *pseudomallei* infection requires optimal responses from both cellular and humoral immune systems [7,8]. IFN-γ secreted by NK cells and T cells plays an important role in the control of infection in mice [9]. To date, one of the main candidate antigens is bacterial flagellin (FliC), which assembles to form the flagellar filament that supports bacterial motility. FliC induces IFN-γ responses from human T cells, and is recognized by antibodies from seropositive individuals living in endemic areas [10,11]. In a mouse model of infection, CpG-modified plasmid DNA encoding flagellin, induced responses from Th1 cells and B cells, and was shown to protect against bacterial re-infection and to reduce mortality and morbidity rate [11]. Furthermore, in passive immunization trials, antibodies raised against FliC from *B*. *pseudomallei* strain 319a were shown to protect diabetic rats challenged with a heterologous *B*. *pseudomallei* strain [12,13].

Interestingly, FliC is recognized as a pathogen associated molecular pattern (PAMP) by Toll-like receptor 5 (TLR5) and by nucleotide-binding oligomerization domain (NOD)-like receptor C4 (NLRC4), activating both innate and adaptive immunity [14,15]. Indeed, both are pattern recognition receptors (PRR), and play a key role in innate immunity, offering a first line of defence against invading pathogens. PAMPs are prevalent in bacterial but not in vertebrate genomic DNA; the immune system appears to exploit these molecules as signaling beacons to reveal the presence of infection and activate appropriate defense pathways [16]. It has been proven that TLR5 interacts with the conserved D1 domain of FliC, responsible for flagellin assembly [17]. Therefore, in addition to the direct stimulation of a protective response, FliC could function as a molecular adjuvant in combination with other specific antigen(s) [18].

With regards to flagellin from *B*. *pseudomallei* (FliC_Bp_), epitope mapping, performed in our laboratory at Khon Kaen University, Thailand by synthesis of 38 multiple overlapping peptides extending along the whole protein sequence was carried out and identified several peptides that bound to HLA class II alleles found in Thailand [19]. This approach, however, is costly and time consuming. Sequence-based and 3D structure-based computational predictions for consensus dominant epitopes represent alternative, inexpensive and rapid approaches for epitope discovery and vaccine development [20].

In this report, we focused on FliC_Bp_ as a target antigen for sequence and structure-based epitope discovery. On this basis, we designed and synthesized the predicted epitopes as free peptides for consideration as potential vaccine components. To this aim, we used three B-cell epitope prediction servers [21,22] and one T-cell epitope prediction server [23], to identify putative *ab initio* immunoreactive linear epitopes. Analysis of the sequence outputs from all servers resulted in the identification of three linear epitopes with predicted B-cell and T-cell activities. The three selected sequences were synthesized as conjugated peptides, and tested for their effective T-cell/B-cell activities in immunological studies.

In parallel, we solved the high-resolution crystal structure of FliC_Bp_ as a starting point for future structure-based antigen and epitope design and optimization that permitted the identification of additional potentially antigenic regions. To this aim, we used a combination of 3D structure-based epitope predictions [24,25] and an experimental epitope mapping technique to broaden our consensus toward the identification of novel epitopes. Upon comparison of sequence-based and structural T-cell predictions, we identified and synthesized an additional T-cell epitope that was also tested in immunological studies.

FliC_Bp_ constitutes a major target for vaccine discovery initiatives to elicit protective immunity against *B*. *pseudomallei* infections. The predicted epitope sequences here-identified, coupled to the structural information about their native conformations, hold great potential for a process of rational design toward the optimization of their antigenic properties, and display the characteristics required for presentation as vaccine components.

## Materials and Methods

### Blood samples

Ethical permission was obtained from the KKU Ethics Committee for Human Research no. HE470506 and HE561234. All subjects were adults and had received written information before signing the consent form. Heparinized whole blood samples were collected from healthy donors, seropositive (IHA titer > 40) and seronegative (IHA titer ≤ 40) individuals [26,27], at the Blood Bank Center, Khon Kaen University, and from recovered melioidosis patients at Srinakarind Hospital, Khon Kaen University, Khon Kaen, Thailand.

### Prediction of B and T cell epitopes using web servers

The full-length FliC_Bp_ protein sequence was submitted to B cell linear epitope predictors, BepiPred (http://www.cbs.dtu.dk/services/BepiPred/) [21]; threshold = 0.35, BCPred (http://ailab.ist.psu.edu/bcpred/predict.html) [22]; classifier specificity = 75%, and AAP (http://ailab.ist.psu.edu/bcpred/predict.html) [22]; classifier specificity = 75%). Any sequences that were positively selected by more than two prediction methods and that contained more than 15 amino acids were selected as linear B cell epitopes. Predicted linear B cell epitopes were then re-submitted for T cell epitope predictions using the MHC-II Binding Predictor (http://tools.immuneepitope.org/mhcii/) in the Immune Epitope Database (IEDB) [23], with HLA DRB1 alleles common to Thailand; HLA DRB1*0301, 0405, 07, 09, 1202, 1501, 1502 and 1602 [28]. Positive T cell epitopes were identified by a HLA binding prediction score of less than 30.

### Recombinant FliC_Bp_ production, crystallization, data collection, and structure determination

The *BPSL3319; fliC* gene encoding for protein residues 25–378 (devoid of the N-terminal signal peptide (1–24) and C-terminal residues 379–388), was amplified from *B*. *pseudomallei* strain K96423 (Kindly provided by Prof. R. Titball, University of Exeter, UK), cloned into pGEX4T1 (Life Technologies) and expressed as a GST-fusion protein in BL21 Star (DE3) *Escherichia coli* cells (Life Technologies) and purified by affinity chromatography on a 5mL GSTrap FF column (GE Healthcare) pre-equilibrated in 1X PBS. On-column cleavage of the GST tag was carried out upon addition of 100 U thrombin (Sigma-Aldrich) in 1X PBS, incubation overnight at RT. Cleaved FliC_Bp_ was eluted with 1X PBS and thrombin was removed using a 1ml HiTrap Benzamidine FF column (GE Healthcare), according to the manufacturer’s instructions. FliC_Bp_ was exchanged into 10 mM Tris-HCl pH 8.0 and concentrated to 10.5 mg/ml for crystallization trials.

FliC_Bp_ crystals were grown by sitting drop at 20°C, in a 300 nl drop containing 50% protein solution (10.5 mg/ml) and reservoir solution (0.1 M HEPES pH 7.5, 25% PEG 6000 and 0.1 M Lithium Chloride). Crystals were cryo-protected in reservoir solution containing 30% glycerol. One crystal was used to collect X-ray diffraction data at the ID23-2 beamline at the European Synchrotron Radiation Facility (Grenoble, France). Data were processed using IMOSFLM and scaled using POINTLESS and SCALA using the CCP4 suite [29,30]. The structure was solved via molecular replacement using Phaser [31] and the structure of domain D1 (residues 62–168 and 281–326) of *Sphingomonas* sp. A1 flagellin (PDB entry 2ZBI) as a search model (the structure of FliC_Pa_ was not available at that time). Residues of the search model not conserved in FliC_Bp_ were substituted with alanines. The experimental phases were improved by applying Arp/Warp [32], and the amino acid sequence of the model was then modified to match the correct FliC_Bp_ sequence. The FliC_Bp_ structure was completed by manually fitting in the electron density residues of the D2 domain, which inserts between residues 167 and 287 of domain D1. Several rounds of manual model building with COOT [33], and refinement with the program REFMAC5 [34], were carried out until refinement reached convergence and the quality of the model was checked with PROCHECK [35]. The final refinement statistics and geometry quality parameters are shown in S1 Table. Atomic coordinates and structure factors were deposited in the Protein Data Bank with PDB code 4CFI [36].

### Epitope prediction and design

The crystal structure of FliC_Bp_ was used as a starting point for three replicas of all-atom Molecular Dynamics (MD) simulations in explicit water at 300K. Each replica was 50 ns long and was carried out in NPT conditions. The simulations and the analysis of the trajectories were performed using the GROMACS 4.54 software package [37],[38], the SPC water model [39] and the GROMOS53A6 force field [40]. The procedure employed is fully described in Gourlay *et al*., [41].

### Matrix of Local Coupling Energies (MLCE) method

Epitope predictions were carried out using MLCE on the representative structure of the most populated structural cluster of each MD trajectory [24]. The clustering procedure was performed using the method developed by Daura *et al* [40]. The MLCE method is based on the calculation of the matrix of inter-residue, non-bonded interaction energies using a MM-GBSA (Molecular Mechanics Generalized Born Surface Area) implicit solvent approximation. The principal components of the interaction matrix are selected by eigenvalue decomposition [42,43,44,45,46] and filtered by the protein contact map to select the substructures presenting low energy couplings with the rest of the protein 3D fold. These substructures are characterized by dynamic properties allowing them to visit multiple conformations, a subset of which can be recognized by the antibody. MLCE is available as the free web tool BEPPE (http://bioinf.uab.es/BEPPE).

### Electrostatic Desolvation Profiles (EDP) method

The EDP method calculates the free energy penalty for desolvation placing a neutral probe at various protein surface positions. Surface regions with a small free energy penalty for water removal may correspond to preferred interaction sites. Evidence suggests that it is easier for an antibody to bind to an epitope when properties required for high affinity binding like low desolvation penalty are met [25].

### *In vitro* epitope mapping

Peptide mixtures were obtained by trypsin digestion of recombinant FliC_Bp_ in 50 mM ammonium bicarbonate buffer (pH 7.8) at a ratio of 10:1, at 37°C for 3 h. To capture the epitope-containing peptide, a 25 μl suspension of Dynabeads Pan Mouse IgG (uniform, super-paramagnetic polystyrene beads of 4.5 μm diameter coated with monoclonal human anti-mouse IgG antibodies) was used. The beads were washed twice with PBS using a magnet and re-suspended in the initial volume. 50 μl of the murine serum were added and incubated for 30 min at room temperature (RT), after which the beads were washed five times with PBS to remove serum debris. 0.5 μl of Protease Inhibitor Mix (GE healthcare) were added before the peptide mixture to avoid potential antibody degradation. The sample was then incubated for 2 h at RT with gentle tilting and rotation. After incubation, beads were washed three times with 1 ml PBS, and the bound peptides were eluted in 50 μl of 0.2% TFA. The elution fraction was concentrated and washed with C18 ZipTips (Millipore) and eluted in 2 μl of 50% ACN and 0.1% TFA. Subsequent MALDI-MS analysis of the eluted fractions was carried out. For more details, see Gourlay *et al*., [41].

### Peptide synthesis

All peptides were manually assembled by stepwise Fmoc-SPPS onto a 2-Chlorotrityl chloride resin (2-CTC), using HBTU/DIEA for *in situ* activation of entering amino acids [47]. Piperidine 20% in DMF was used for Fmoc removal steps. Cysteine was added to the N- of peptides to enable specific conjugation to carrier proteins, using poorly immunogenic PEG units as spacers. Upon completion of peptide assembly, peptides were simultaneously cleaved from the resin and side chains were deprotected by treatment with a mixture of 2.5% water, 2.5% thioanisole, 2.5% ethanedithiol, 2.5% triisopropylsilane and 90% trifluoroacetic acid for 2 hours at RT. Crude peptides were precipitated in cold diethyl ether, collected by centrifugation and washed with further cold ether Peptides were subsequently dissolved in 50% aqueous ACN/0.1% TFA and purified by C18-RP-HPLC. Peptide purity and identity was assessed by analytical C18-RP-HPLC and separate ESI-MS analysis.

### Peptide conjugation to carrier proteins and polyclonal anti-peptide preparation

Peptide N-terminal cysteine residue, preceded by the PEG spacer, allowed selective conjugation to carrier proteins (human serum albumin, (HSA), Hemocyanin from Concholepas (KLH) and Rabbit Serum Albumin (RSA)) using sulfosuccinimidyl 4-(N-maleimidomethyl) cyclohexane-1-carboxylate (Sulfo-SMCC) bifunctional linker. All peptides were prepared in free- and conjugated forms. Polyclonal antibodies were raised against peptide epitopes in rabbits (Primm srl, Milano Italy). Antisera against the FliC_Bp_ peptides were immunopurified against the peptides chemically linked to Cyanogen Bromide Activated Sepharose (Sigma-Aldrich).

### Detection of rabbit or human antibodies (IgG) in serum by indirect ELISA

Rabbit or human antibody recognition was detected as previously described [41]. In brief, 96-well polystyrene plates (Nunc Maxisorp) were uncoated or coated with 50 μl/well of 1 μg/ml of *B*. *pseudomallei* K96243 crude extract (crude Bps), 3 μg/ml of FliC_Bp_ protein or HSA-conjugated predicted peptides or HSA in 0.1 M carbonate-bicarbonate buffer (pH 9.6), and incubated at 37°C for 3 hr. After washing, 50 μl/well of 1:300 diluted human plasma or 1:3,000 rabbit antisera samples were probed in duplicate. Immunoreactivity was detected and represented as absorbance index of individual samples = (O.D._test_−O.D._uncoated_) / O.D._uncoated_.

### *In vitro* human peripheral blood mononuclear cell (PBMC) stimulation and cytokine detection by indirect ELISA

PBMCs from each donor were isolated by density gradient centrifugation on Ficoll-Hypaque and stored at -80°C with 10% dimethyl sulfoxide (DMSO) in fetal bovine serum (FBS). Immediately prior to the experiment, frozen PBMCs were thawed and resuspended in RPMI 1640, supplemented with 10% FBS, 200 U/ml penicillin, and 200 mg/ml streptomycin. 5 x 10^5^ PBMCs/well were plated in 96-well culture plates and cultured with fixed Bps (PBMCs:organism = 1:30), 3 μg/ml of phytohaemagglutinin (PHA), 10 μg/ml of recombinant FliC_Bp_, and 50 g/ml peptides for 48 h. Gamma interferon (IFN-γ) and interleukin 10 (IL-10) levels in the supernatant were quantified using a cytokine detection ELISA kit (BD biosciences) according to the manufacturer’s instructions. The sample with a detectable cytokine level higher than the lower limit of detection (>45 pg/ml) was described as responder.

### Polymorphonuclear (PMN) cell phagocytosis and oxidative burst assay

Human PMN cells were purified by 3.0% dextran T-500 sedimentation and Ficoll-PaquePLUS centrifugation (Amersham Biosciences), as previously described [41]. Generally, PMN cell purity was >95%, as determined by Giemsa staining and microscopy; viable cells were counted by trypan blue exclusion (viability >99%). 1 x 10^9^ CFU/ml of intact *B*. *pseudomallei* K96243 cells were labeled with 1 mg/ml of FITC in the dark at room temperature for 1 h. Intensity FITC on labeled bacteria was measured by flow cytometry. 7.5 x 10^7^ CFU/ml FITC-labeled bacteria were treated with medium alone or antibody at concentrations of 40 μg/ml, for 1 h at 37°C, before being cultured with purified human PMN cells for 15 min at 37°C. 1:50 rabbit anti-FliC_Bp_ antisera was used as positive control for phagocytosis assay, while, 800 ng/ml of PMA (Sigma-Aldrich) was used as a positive control for the oxidative burst assay. Subsequently, 25 μl of 2,800 ng/ml of hydroethidine (HE) (Sigma-Aldrich) was added and incubated for 5 min at 37°C, washed twice and fixed with 2% paraformadehyde. Phagocytosis of FITC labeled Bps+ and oxidative burst activities of PMN cells that turn hydroethidine (HE) to ethidium bromine (EB+) were analyzed by flow cytometry (FACSCalibur; BD Biosciences). Results are represented as % phagocytosis (total % FITC+) and % oxidative burst (% EB+ FITC+) (details shown in S1 Fig). The differences between antibody groups were tested by the paired t test.

### Intracellular bacterial killing assay

Live *B*. *pseudomallei* K96243 was opsonized, with or without 1 μg/ml of anti-FliC_Bp_ peptide antibodies, as described above. Rabbit pre-breed serum at 1:50 was used as negative control while rabbit anti-FliC_Bp_ antiserum at 1:50 was used as positive control.

Purified human PMN cells at 5 x 10^5^ cells were incubated with opsonized bacteria (multiply of infection; MOI = 10) for 30 min at 37°C. Extracellular bacteria were subsequently killed upon addition of 250 μg/ml of kanamycin for 30 min, at this time point; PMN cells were lysed and plated on LB agar for bacterial counts (T0). In another condition, 20 μg/ml of kanamycin was added to maintain extracellular bacteria and incubated for 3 h at 37°C, and bacteria numbers were counted (T3).

## Results

### Sequence-based prediction of FliC_Bp_ B- and T-cell linear epitopes

**Fig 1:**
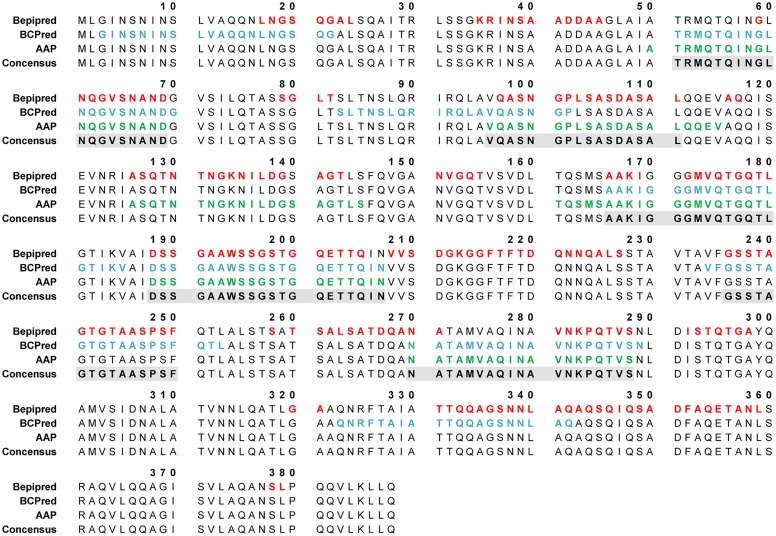
FliC_Bp_ B-cell linear sequence-based epitope predictions using three predictor methods: BepiPred (threshold = 0.35), BCPred (classifier specificity = 75%), and AAP (classifier specificity = 75%). Epitope predictions were carried out on the full-length amino acid sequence of FliC_Bp_, using three web-accessible prediction servers: BepiPred, BCPred and AAP [21,22]. Predicted epitope residues by BepiPred, BCPred and AAP are shown in red, blue and green font, respectively on the amino acid sequence (residue numbers are indicated). Grey shaded boxes indicate consensus positive residues identified by at least two epitope predictors.

Epitope predictions were carried out on the full-length (residues 1 to 388) amino acid sequence of FliC_Bp_ (UNIPROT code H7C7G3), using the web-accessible prediction servers BepiPred, BCPred and AAP [21,22]. Six linear B cell epitopes were identified (Fig 1) and subsequently used for T cell epitope predictions using the IEDB server [23,48] (www.iedb.org), selecting for HLA DRB1 alleles common in Thailand; HLA DRB1*0301, 0405, 07, 09, 1202, 1501, 1502 and 1602 [28]. A detailed overview of the consensus between linear epitopes predicted by sequence-based web servers is shown in Fig 1. The top three epitopes that were predicted to be both B and T cell epitopes are F51-69, F96-111 and F270-288. Interestingly, F51-69 overlaps with Peptide 6 (residues 51–70); F96-111 overlaps with Peptide 10 (residues 91–110), and F270-288 overlaps with Peptide 28 (residues 271–290) from the peptide panel synthesized by Musson et al. The following peptides were synthesized as described in the Materials and Methods section: F51-69; 51-TRMQTQINGLNQGVSNAND-69, F96-111; 96-VQASNGPLSASDASAL-111, F270-288; 270-NATAMVAQINAVNKPQTVS-288.

### Predicted FliC_Bp_ epitope peptides, F51-69 and F270-288 are recognized by human antibodies (IgG) in serum, and also elicit responses from human PBMCs

**Fig 2:**
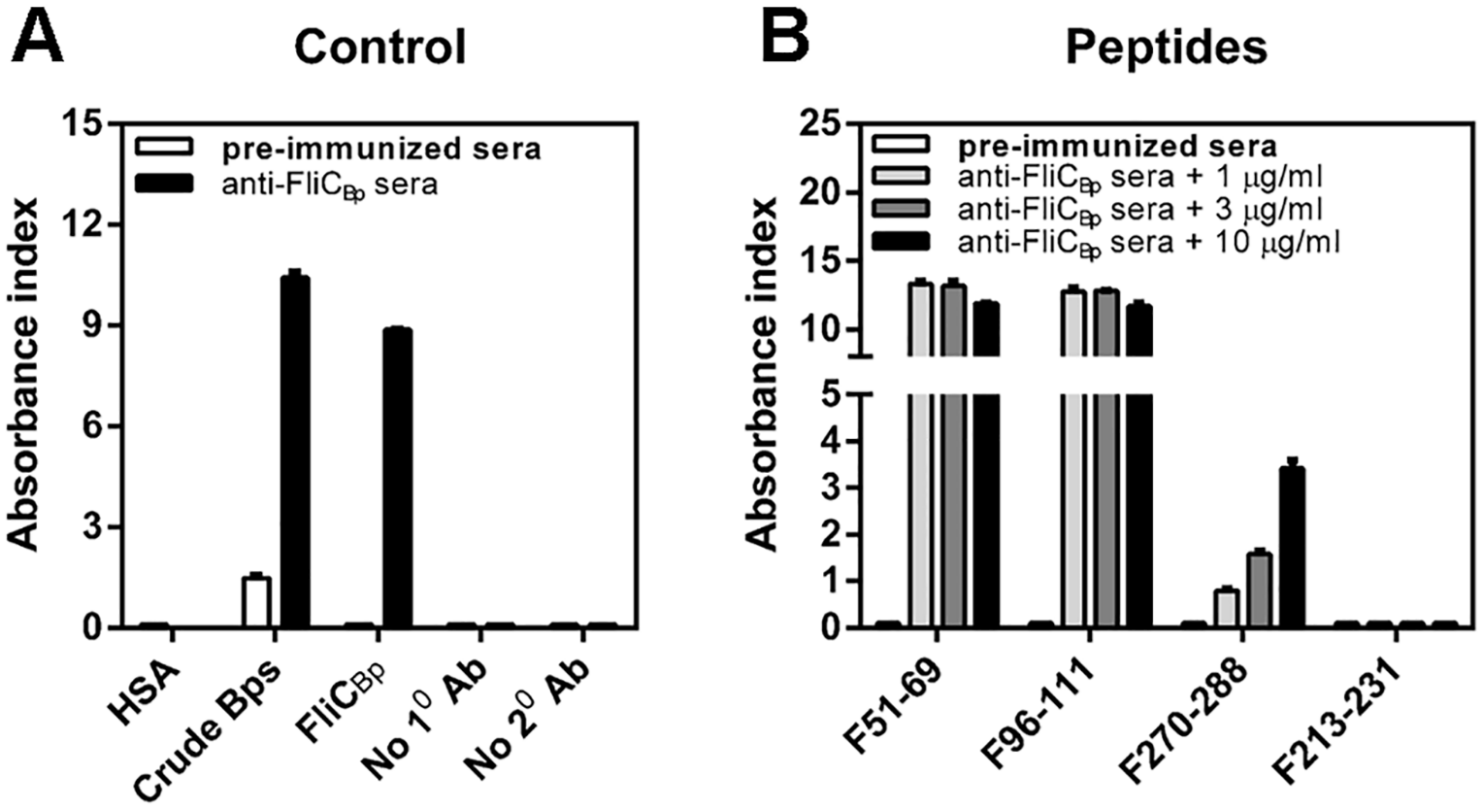
Recognition of predicted epitope peptides by rabbit anti-FliC_Bp_ sera as detected by Indirect ELISA. Controls (A) composed of human serum albumin (HSA), crude *B*. *pseudomallei*, recombinant FliC_Bp_, without primary antibody (No 1^0^ Ab) and without secondary antibody (No 2^0^ Ab) and F51-69, F96-111, F270-288 and F213-231 peptides (B) were coated at various concentration (1, 3 and 10 μg/ml) onto a 96-well polystyrene plate and probed with diluted rabbit antibodies to FliC_Bp_ and quantified by indirect ELISA. Results are represented by the Absorbance index (O.D._test_−O.D._uncoated_ / O.D._uncoated_). Experiments were performed in duplicate and results represent the mean of the Absorbance index ± SE.

**Fig 3:**
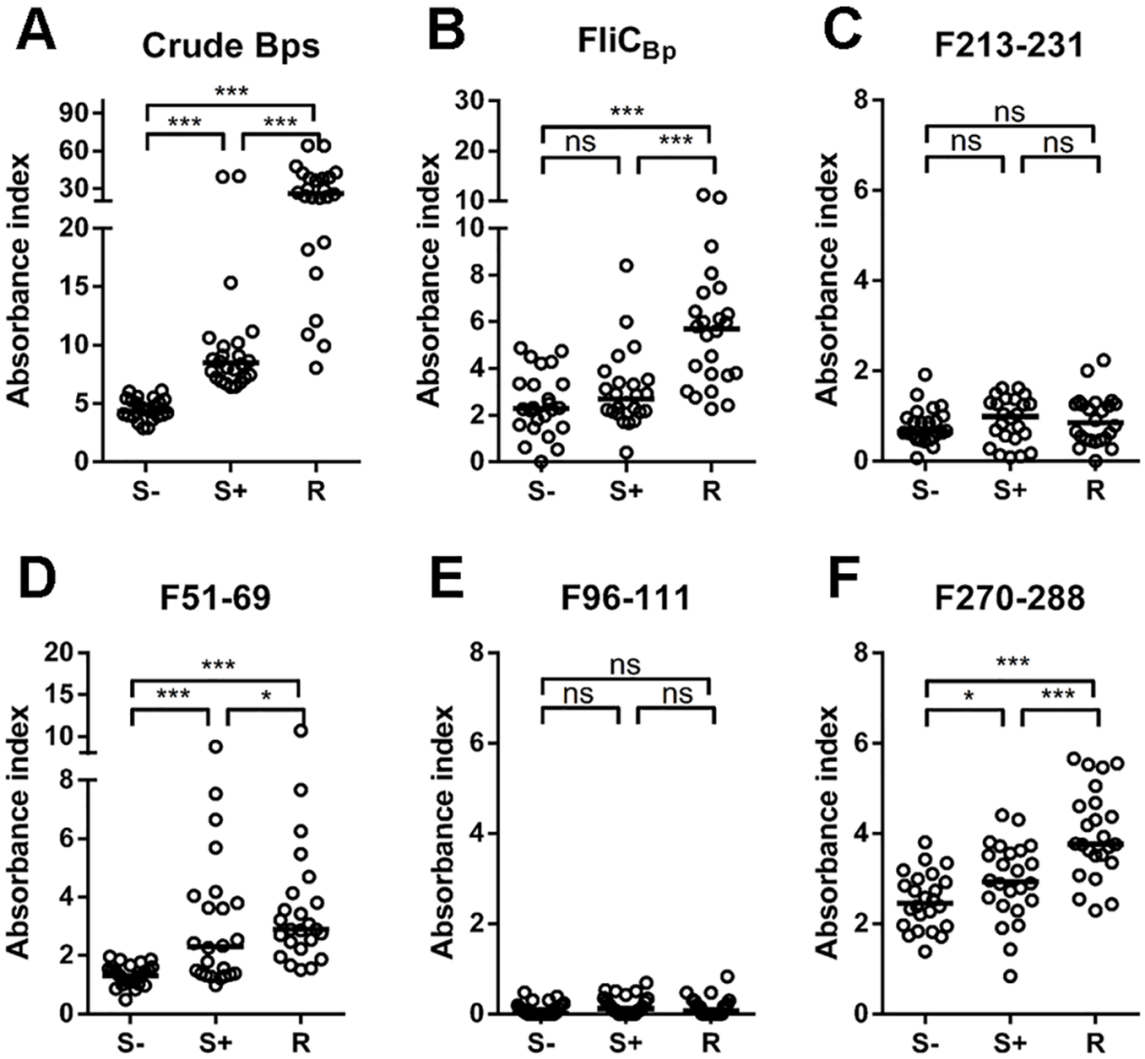
Distribution of human antibody against *B*. *pseudomallei* related proteins and peptides among seronegative (S-; n = 24), seropositive (S+; n = 24) and melioidosis recovered individuals (R; n = 24), detected by Indirect ELISA. Crude *B*. *pseudomallei* antigens (A) recombinant FliC_Bp_ (B), FliC_Bp_ peptide F213-231 (C), F51-69 (D), F96-111 (E), F270-288 (F) were coated onto a 96-well polystyrene plate and probed with diluted plasma samples from healthy and recovered individuals and quantified by indirect ELISA. Results are represented by the Absorbance index (O.D._test_−O.D._uncoated_ / O.D._uncoated_). Experiments were performed in duplicate and results represent the mean of the Absorbance index ± SE. * *P* < 0.05, ** *P* < 0.01, *** *P* < 0.001, ns = not significant compared between plasma sample groups using one-tailed Mann-Whitney U test.

Predicted B and T cell epitopes were initially evaluated for immunorecognition by probing them with rabbit anti-FliC_Bp_ anti-sera. All three peptides were recognized (Fig 2). Subsequently, crude *B*. *pseudomallei* extract (Bps), recombinant FliC_Bp_, and peptides F51-69, F96-111 and F270-288 were tested against human plasma samples for immunorecognition (Fig 3). In order to investigate a possible role of the peptides in protection, samples were tested from diverse sub-populations, including healthy seronegative, healthy seropositive and recovered melioidosis patient groups. Results showed that F51-69 and F270-288 were recognized by antibodies in human plasma, in contrast to F96-111. In addition, antisera from recovered individuals were shown to recognize Bps extract, FliC_Bp_, and all peptides, except for F96-111, to a greater extent than those from healthy seropositive and seronegative individuals (Fig 3).

Moreover, upon comparison of antibody reactivity between healthy sample groups, we found that the levels of reactivity to crude Bps, F51-69 and F270-288 in the seropositive group were significantly higher than those in the seronegative group, p < 0.001, p < 0.001 and p < 0.05, respectively (Fig 3B). In case of FliC_Bp_, there was a background of antibodies in seronegative group that might be due to *B*. *thailandensis* flagellin as it shares 91% similarity to *B*. *pseudomallei* flagellin compared by Basic Local Alignment Search Tool (BLAST). However, the levels of antibody against FliC_Bp_ in melioidosis recovered group were significantly higher than both seronegative and seropositive groups. Taken together, this suggests that people who are exposed and/or infected with *B*. *pseudomallei* develop antibodies against FliC_Bp_, and more specifically against F51-69 and F270-288 epitopes (Fig 3). In contrast, peptide F96-111 was not recognized in human samples, despite high reactivity against rabbit anti-sera. This is likely due to the fact that this peptide is accessible in the recombinant protein, but not when FliC_Bp_ is assembled in the flagella, as supported by the observation that antibodies raised against F96-111 do not recognize crude *B*. *pseudomallei* (S2B Fig).

An additional confirmation of antibody specificity was illustrated by coating ELISA plates with crude Bps extract and each synthesized peptide. Each plate was probed with human plasma samples, previously neutralized with the same extract/peptide at different concentrations (S3 Fig). According to this test, peptides F51-69 and F270-288 inhibited the activity of antibodies in the recognition of their corresponding sample coated on the plate in a dose-dependent manner (S3 Fig).

Following our analysis of the FliC_Bp_ crystal structure (see below) and comparison with FliC from *S*. *typhimurium*, it became apparent that F96-111 is located in a region of the protein that mediates monomer-monomer interactions during assembly of FliC into its 11-subunit ring protofilament arrangement [49]. Therefore, when FliC_Bp_ is assembled in its native form in the flagella, this region would not be solvent accessible, in agreement with its lack of recognition by human antibody.

**Fig 4:**
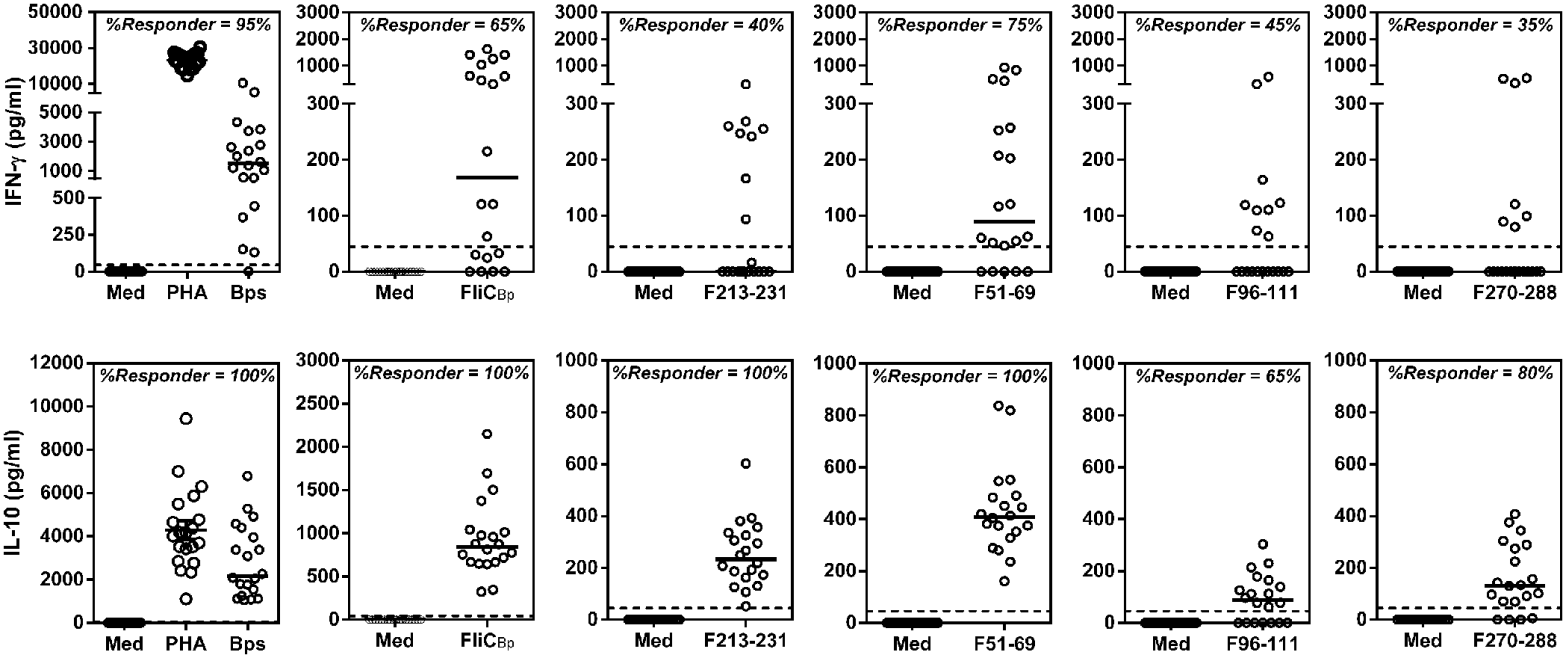
FliC_Bp_ peptides induce human IFN- and IL-10 production from PBMCs. PBMCs (5 x 10^5^ cells/well) from 20 seropositive healthy donors were stimulated with culture medium alone (Med), fixed Bps (PBMCs:organism = 1:30), 3 μg/ml of phytohaemagglutinin (PHA), 10 μg/ml of FliC_Bp_ protein, and 50 g/ml of FliC_Bp_ peptides (F51-69, F96-111, F270-288 and F213-231) for 48 h, and the IFN- and IL-10 levels were quantified by ELISA. Vertical lines represent the median and dashes indicate the detection limit.

To further our studies, we then assessed whether the peptides could be active as human T cell epitopes, as implied by the bioinformatics predictions. Peptides were cultured with healthy PBMCs for 48 h, and the culture supernatants were measured for IFN-γ and IL-10 production using ELISA, as described in the Materials and Methods. We found that almost all samples (19/20; 95%) responded to intact killed Bps via IFN-γ production, some responded to FliC_Bp_ (13/20; 65%), F51-69 (15/20; 75%), F96-111 (9/20; 45%) and F270-288 (7/20; 35%), as shown in Fig 4. According to our results, FliC_Bp_ protein and predicted epitope peptides, in particular F51-69, elicited IFN-γ production from human PBMCs. With regards to an IL-10 response, all samples responded to fixed Bps, although with variable intensity. Recombinant FliC_Bp_ and peptide F51-69 responded in all samples (100%), however only 13/20 samples responded to F96-111 (65%) and 16/20 samples to F270-288 (80%). By combining the results of immunoreactivity against patient sera and the stimulation of cytokine production, we may confirm that epitopes F270-288 and F51-69, in particular, are promising B cell and T cell epitopes.

### Antibodies against FliC_Bp_, F51-69 and F270-288 enhance phagocytosis, oxidative burst and bacterial killing in primary human neutrophils

**Fig 5:**
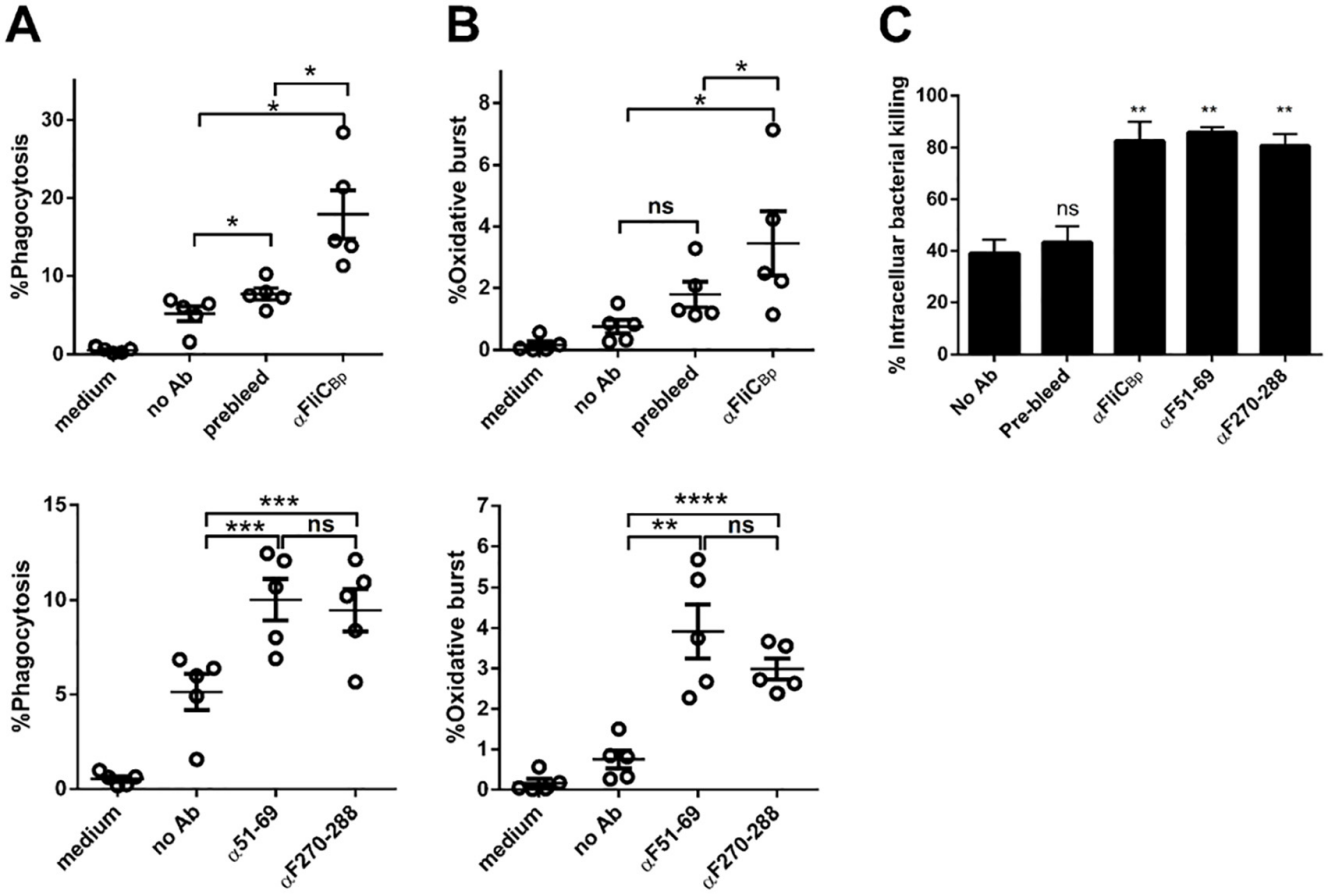
Rabbit anti-FliC_Bp_ predicted peptide antibodies enhance phagocytosis (A), oxidative burst (B) and bacterial killing (C) against *B*. *pseudomallei* in purified human PMNs (N = 5). Rabbit anti-FliC_Bp_, pre-bleed anti-sera at dilution 1:50 or anti-predicted peptide antibodies at 40 g/ml were used for opsonization. * *P* < 0.05, ** *P* < 0.01, *** *P* < 0.001, ns, not significant compared between with or without antibody groups using one-tailed paired t test.

Our results show that antibodies against F51-69 and F270-288 are present in plasma of healthy donors who have been exposed to *B*. *pseudomallei*. Therefore we aimed to investigate whether these antibodies may stimulate phagocytosis by neutrophils and induce bacterial killing. Rabbit polyclonal antibodies were raised against F51-69 and F270-288 peptides (see Materials and Methods). Prior to experiments, the activity of antibodies against intact *B*. *pseudomallei*, recombinant FliC_Bp_ and peptides were checked by indirect ELISA. Results showed that all rabbit antisera recognized intact *B*. *pseudomallei*, recombinant FliC_Bp_ and immunized peptides (S3 Fig). Antibodies were subsequently used in *B*. *pseudomallei* opsonization tests and analyzed for bacterial phagocytosis and oxidative burst production in human neutrophils. Results revealed that antibodies raised against recombinant FliC_Bp_ and predicted epitope peptides enhance both phagocytosis and oxidative burst activity in human neutrophils (Fig 5A and 5B). Interestingly, we also found that these antibodies also enhance bacterial uptake and intracellular bacterial killing in human neutrophil infection studies (Fig 5C). These results suggest that antibodies against F51-69 and F270-288 may enhance host resistance to *B*. *pseudomallei* infection by stimulating neutrophil phagocytosis and bacterial killing.

### 3D structural analyses of FliC_Bp_ by X-ray crystallography

FliC_Bp_ (residues 25 to 378) was crystallized using the sitting drop vapor diffusion method, as described in the Materials and Methods section. The crystal structure of FliC_Bp_ was solved at a resolution of 1.3 Å by molecular replacement, using the structure of domain D1 (residues 62–168 and 281–326) of *Sphingomonas* sp. A1 flagellin (PDB entry 2ZBI) as an initial search model, and refined to satisfactory R_gen_ and R_free_ values of 13.6% and 16.4%, respectively (statistics for the data collection and model refinement are shown in S1 Table). Electron density was visible for residues 69–326; the first 44 N-terminal residues and the 52 C-terminal residues were absent, however experimental epitope mapping revealed two immunocaptured peptides containing N-terminal residue 37 and residue 361, therefore the residues that are not visible in the electron density are likely to be present in disordered regions of the protein.

**Fig 6:**
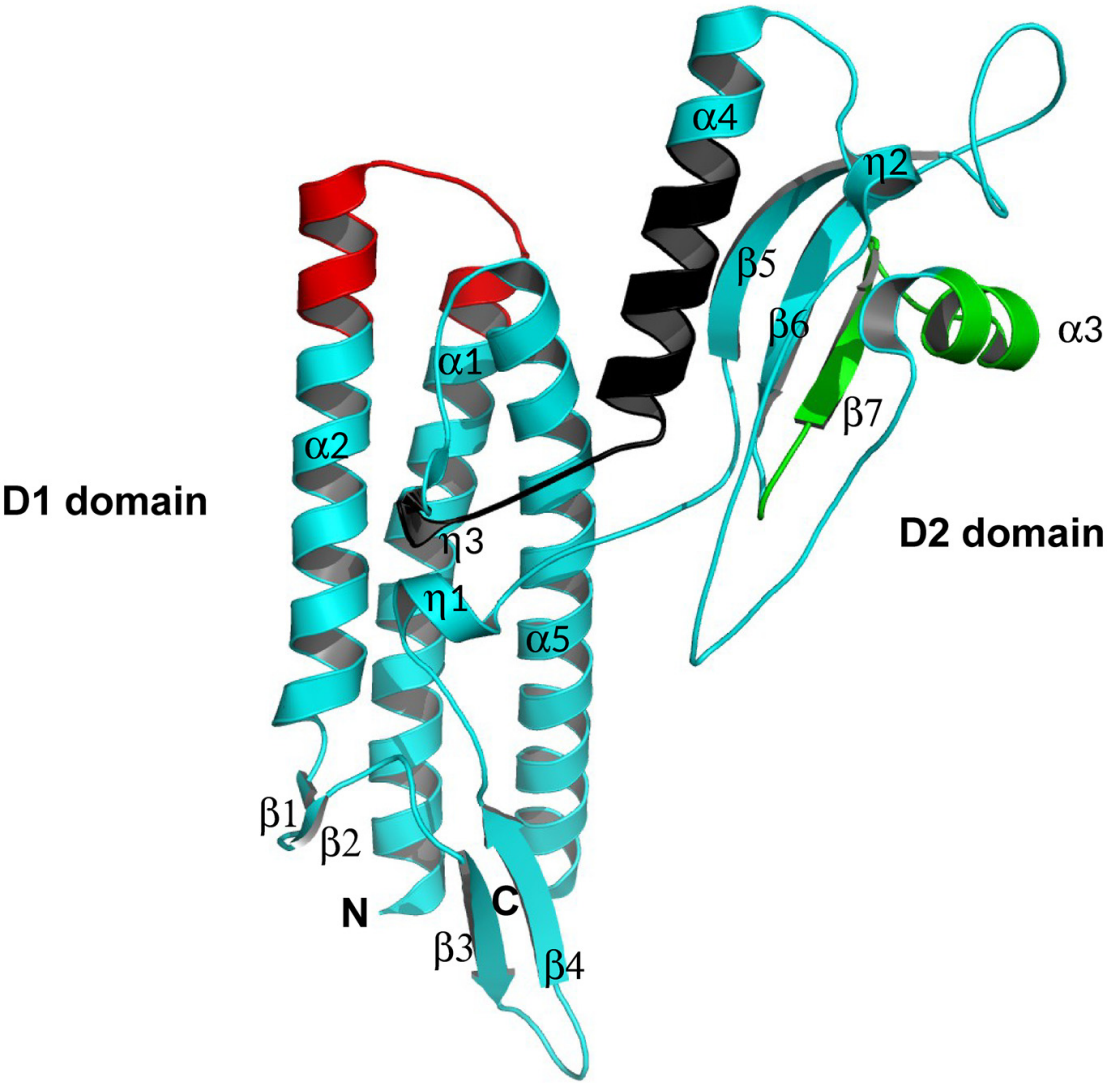
Domain organization and 3D crystal structure of FliC_Bp_. Schematic secondary structure ribbon representation of the crystal structure of FliC_Bp_. The N- and C-termini are labeled as are the diverse secondary structural features with α-helices in cyan and β-strands in magenta. Peptides 96–111, 213–233 and 270–288 are highlighted in red, green and black, respectively. This figure was produced using MacPymol.

Flagellins vary in dimension (28–65 kDa) and contain at least two essential highly conserved domains, D0 and D1 that mediate assembly of the flagellar filament, and may contain a second (D2) or third (D3) variable domain. Typically, removal of the D0 domain is necessary for successful crystallization. This was not the case for FliC_Bp_ where, however, the first 44 N-terminal residues (corresponding to the D0 domain) are not visible in the electron density map because of structural disorder. Overall, FliC_Bp_ adopts the canonical flagellin fold and presents two domains, a conserved D1 domain (residues 69–167, 287–326), and a variable D2 domain (168 and 286) (Fig 6). D1 forms a helical rod (α1, α2 and α5) involving residues contributed by both the N-terminus (residues 69–170) and C-terminus (residues 285–326), a β-hairpin domain (β1-β2) and a 3_10_ α-helix (η1). Domain D2 (residues 172–287) contains a three-stranded antiparallel β-sheet, two α−helices (α3 and α4) and two 3_10_ α-helices (η2 and η3). Domain D2 is connected to D1 *via* two anti-parallel regions that house two short 3_10_ α-helices (η1 and η3) that interact with each other, thus stabilizing the relative positions of the two domains (Fig 6).

The closest structural homolog to FliC_Bp_ available in the Protein Data Bank is flagellin from *Pseudomonas aeruginosa* (FliC_Pa_; PDB entry 4NX9) [50], with whom it shares 36.5% sequence identity and 96.2% secondary structure similarity and a *rms* deviation of 1.34 Å over 168 aligned Cα pairs (S4 Fig). The structural similarity is limited to the D1 domains, while domain D2 is different in FliC_Bp_ and FliC_Pa_. The main difference arises due to the 7 β-strands present in the D2 domain of FliC_Pa_ in comparison with three present in FliC_Bp_ (S4 Fig). Furthermore, due to the diverse tertiary positioning of the D2 domains, when the D1 domains are aligned, the D2 domains do not superimpose (S4 Fig).

Both D1 and D2 domains have been shown to be important for innate immune recognition [51,52]. With regards to the location of the predicted epitope peptides on the 3D structure, F51-69 could not be mapped onto the structure as electron density was absent for this region, indicating that it is likely to be non-structured. F96-111 is housed in domain D1 and is composed of two short α-helical segments at the C- and N- termini of α-helices 1 and 2, connected by a short loop.

The third predicted peptide F270-288, is located in domain D2 and is formed by α-helix 4, the 3_10_ α-helices η3, and their connecting loop region (Fig 6).

### Structure-based prediction of FliC_Bp_ B- and T-cell epitopes via MLCE and EDP methods

In the context of a Structural Vaccinology project, structure-based computational epitope predictions have proved successful for the identification and design of *B*. *pseudomallei* epitopes with improved immunological properties in comparison with their cognate recombinant proteins, commencing from the crystal structures of the target antigens [41,53].

In order to expand the investigation on FliC_Bp_ beyond the reach of the bioinformatics sequence-based predictions, and possibly complementing it, we employed two structure-based computational methods for epitope identification, and compared the results with the previous consensus. The newly solved 3D structure of the antigen, allows taking a different perspective on epitope discovery. Here, two complementary computational methods for epitope prediction were combined and applied to representative structures obtained from molecular dynamics (MD) simulations run on the FliC_Bp_ crystal structure (see Materials and Methods) [54]. MLCE selects antigenic B- and T-cell epitopes while the EDP identifies general protein-protein interaction interfaces. Predictions produced individually by MLCE and EDP are aligned with the sequence-based consensus and presented in S5 Fig.

### Experimental B-cell epitope mapping

Epitope mapping experiments were carried out using recombinant FliC_Bp_ and cognate polyclonal sera. To this aim, we adopted and extended an immunocapturing approach successfully used previously with monoclonal antibodies [55]. The approach involves proteolytic digestion (using diverse proteases) of the target antigen prior to immunocapturing, and subsequent analysis of antibody-bound peptides (containing epitopes or fragments thereof) by mass spectrometry. Using trypsin for the partial digestion of FliC_Bp_, 5 different peptides were captured by the polyclonal IgGs and subjected to MS/MS analysis: a 1529.807 Da peptide corresponding to the N-terminal segment 37-INSAADDAAGLAIATR-52 (1529.6 Da); a peptide of 2231.122 Da corresponding to 300-QAMVSIDNALATVNNLQATLGAAQNR-325 (2685 Da); two 2771.356 Da and 3310.671 peptides Da represented the same region of the protein with segment 98-ASNGPLSASDASALQQEVAQQISEVNR-124 (2770.9 Da) and 93-QLAVQASNGPLSASDASALQQEVAQQISEVNR-124 (3310.5 Da), and a 3782.885 Da peptide corresponding to 326-FTAIATTQQAGSNNLAQAQSQIQSADFAQETANLSR-361 (3783 Da) (S5 Fig).

### Selection of an additional T-cell epitope and *in vitro* characterization

Overall, the addition of experimental mapping, as well as EDP and MLCE analysis confirm the accuracy of the previous results, being among the areas of the alignment displaying the best consensus (S5 Fig). The other putative active epitopes, consisting of the overlap of two or more methods, include fragments V148-T155; D188-T203; G236-F250 and L309-F326. According to the new consensus, these sequences represent good candidates for further tests of immune recognition, and are currently being investigated.

Among the activities presented in this communication, we selected an additional T cell epitope to be tested, based on the consensus between IEDB (sequence based) (Table 1) and MLCE (structure-based) (S5 Fig), both having specificity for T-cell epitopes. The linear stretch K213-V231 is not selected by any other B cell specific prediction method, and may be an interesting candidate to be assessed for T cell activation. In the 3D FliC_Bp_ structure, this peptide mainly is formed by α-helix 3 and β-strand 7 that pertain to Domain 2 (Fig 6). We synthesized the new epitope F213-231 and repeated the experiment of PBMC stimulation for cytokine production (Fig 4). This peptide epitope was shown to stimulate IFN-γ production in 8/20 samples (40%) and IL-10 production in all 20 samples tested (100%). These results correlate with our previous results that show that this peptide segment can elicit IFN-γ [19]. In parallel, we assessed immune sera recognition of epitope F213-231 and observed that it is not seroreactive, suggesting that it is a potential T-cell epitope but not a B cell epitope (Fig 3C).

**Table 1:** Analysis of predicted B cell FliC_Bp_ epitopes as potential human T cell epitopes using the HLA binding predictor from Immunoepitope Database (IEDB), focusing on common HLA-DRB1 types in the Thai population.

	Predicted sequences	HLA binding predicted score (DRB1)								
		03:01	04:05	07:01	09:01	12:02	14:01	15:01	15:02	16:01
F51-69	^51^TRMQTQINGLNQGVSNAND^69^	53.34	**23.03**	70.21	50.47	54.56	65.29	30.06	**20.71**	69.72
F96-111	^96^VQASNGPLSASDASAL^111^	**15.32**	**23.79**	38.89	**21.31**	83.10	88.80	52.17	**19.15**	90.09
F166-180	^166^AAKIGGGMVQTGQTL^180^	70.94	51.67	66.32	61.16	80.81	87.30	36.27	28.49	80.25
F189-207	^189^DSSGAAWSSGSTGQETTQIN^207^	70.94	49.39	66.27	52.87	97.57	98.29	75.97	30.22	94.40
F236-250	^236^GSSTAGTGTAASPSF^250^	70.15	47.07	53.65	61.16	95.21	98.44	68.85	34.99	92.62
F270-288	^270^NATAMVAQINAVNKPQTVS^288^	**21.55**	**12.28**	52.37	30.81	**26.30**	**22.33**	**22.39**	**18.87**	41.22
F213-231	^213^KGGFTFTDQNNQALSST^231^	30.37	**29.68**	44.85	45.27	78.14	53.65	48.85	**26.26**	63.35

## Discussion

In the melioidosis vaccine development field, several protein antigens have been tested for their ability to trigger a protective immune response in animal models of disease, however, protection has proven to be limited in all cases [7]. In addition to the adaptive immune response, innate immunity also plays an important role in resistance to *B*. *pseudomallei* infection [3,56]. Neutrophils are one of the key players in cellular immunity that stimulate bacterial killing, both directly or indirectly via cytokine production [57,58]. Therefore, it is likely that a future melioidosis vaccine should comprise antigens/epitopes that trigger both arms of the immune system.

Antigen design guided by computational biology is at the forefront of vaccine development, focusing on the design of only immunogenic portions of the antigen that may be domains or even linear peptides. In fact, epitopes may display elevated antigenic activities in comparison with their parental antigen, as previously observed for two acute phase antigens from *B*. *pseudomallei* [41,53].

In this context, we focused on flagellin from *B*. *pseudomallei* as a target for epitope discovery and design, using both sequence-based and 3D structure based approaches. Flagellins from diverse bacteria have been studied as subunit vaccine candidates; among these, *S*. *typhimurium* FliC induces responses from Th2 in primary immunization tests, followed by Th1-dependent responses that occur later during subsequent infection, which leads to bacterial clearance [59]. Furthermore, protective antibody production against *B*. *pseudomallei* infection has also been reported for mice immunized through a modified plasmid encoding the *fliC* gene, combined with CpG oliogodeoxynucleotide [11]. Several immunogenic epitopes have been identified from FliC homologs, e.g. FliC from *Borrelia burgdoferi* [60] and FliC from *Salmonella typhimurium*, from which four epitopes were identified as CD4^+^ specific T cells epitopes [61,62].

Using sequence-based computational predictions, we identified and synthesized three peptides (F51-69, F96-111 and F270-288) with predicted T-cell and B-cell stimulatory activities. Two peptides (F51-69 and F270-288) were in fact found to be B-cell epitopes and were reactive against human antibodies (IgG) isolated from melioidosis-infected subjects and healthy seropositive subjects. The third peptide (F96-111) was not reactive with sera and, based on 3D-structural considerations, and in light of the crystal structure here presented, it is unlikely to be accessible to antibodies when FliC is assembled in the flagella. In fact, structural comparisons of FliC_Bp_ and *S*. *typhimurium* flagellin (FliC_St_), for which several structural and flagellin assembly studies have been carried out, indicate that F96-111 is housed in a key site that in FliC_St_ mediates axial interactions and protofilament assembly, being also responsible for TLR5 recognition of the monomeric protein [63,64]. As only monomeric FliC activates TLR5, it has been suggested that cellular recognition of FliC requires flagella degradation, for example via phagocytosis or other depolymerization mechanisms [63]. Such an interface location for F96-111 in the assembled protofilament structure was confirmed by the inability of anti-F96-111 antibodies to recognize crude *B*. *pseudomallei* extract, and accounts for the fact that the epitope was not recognized by human antibodies. Thus, while it remains a possible T-cell epitope, F96-111 is unlikely to be a B-cell epitope in FliC_Bp_ oligomeric native state.

Based on sequence alignment with FliC_St_ for which residues involved in intermolecular flagellin contacts are known [63,64], F51-69 should also be part of the assembly interface, and should be buried in the flagella, thus inaccessible to antibodies. However, residues 51–69 are absent in the FliC_Bp_ structure, suggesting that they are structurally disordered, in contrast to equivalent residues from FliC_St_ that instead pertain to the N- terminal α-helix, suggesting that the assembly interfaces may differ between the species. In fact, the F51-69 peptide is recognized by human antibodies, demonstrating that this region is accessible in the flagella and not buried as in FliC_St_.

With regards to the cell-mediated response, our results show that all predicted T/B cell FliC_Bp_ epitopes (in particular F51-69) induce cytokine production (IFN-γ and IL-10) from human PBMCs, and that their cognate antibodies also enhance human PMN phagocytosis and bacterial killing by increasing oxidative burst activity.

We also report the 1.3Å resolution FliC_Bp_ crystal structure as the basis for *in silico* epitope predictions using two diverse computational methods. Our approach, besides allowing us to cross-validate sequence-based and 3D-structure-based epitope prediction methods (resulting in the identification of consensus epitope regions), identified a fourth epitope (F213-231) that, when synthesized as a peptide, was found to be a T-cell epitope but not a B-cell epitope. Significantly, the *in silico* (structure and sequence-based) predictions were complemented by *in vitro* epitope mapping that identified residues predicted by both sequence-based and structure-based methods.

As previously mentioned, it is likely that several protective antigens will be required to formulate an effective protective vaccine [7,65]. Previous experimentation on mice immunization showed that the addition of adjuvants is often necessary in combination with subunit vaccine candidates to elicit protection [65]. TLR agonists are widely used as adjuvants, including flagellin, which is a TLR5 agonist and a potent activator of both the innate and adaptive immune responses [18,50]. This implies that the potential of the T-cell/B-cell FliC_Bp_ peptides identified in this study could represent adjuvants to be administered with other antigen candidates to develop a protective melioidosis vaccine. In fact, a truncated form of FliC_St_ was shown to induce a significantly stronger cellular immune response when administered in a chimeric subunit vaccine together with *Eimeria tenella* immune mapped protein-1 (EtIMP1), in comparison to Freund’s Complete Adjuvant [66]. An additional successful application of FliC as a carrier protein was reported for the glycoconjugate subunit vaccine that improved protection against fetal infection disease [67].

In conclusion, our results shows that both sequence-based and 3D-structure-based epitope discovery methods, and the derived consensus sequence segments, proved successful in the identification of both T-cell and B-cell epitopes from FliC_Bp_. Validatory *in vitro* epitope mapping and immunological tests fully confirmed the predictive analyses. Taken together, our data suggest that the predicted peptide epitopes with confirmed T-cell/B-cell activities should proceed to further *in vivo* immunological tests as potential vaccine components, possibly in conjunction with other known *B*. *pseudomallei* antigens. To this aim, further analyses of additional peptides suggested by the 3D-structure-based predictions will be undertaken to enrich the repertoire of potential protective epitopes.

## Supporting Information

S1 Fig Phagocytosis and oxidative burst assay gating strategy and analysis. Purified PMN cells were gated on the basis of their FSC/SSC. All gates were identical in all samples within each experiment. The degree of phagocytosis and oxidative burst is shown by fluorescent intensities of FL1-FITC and FL2-EB, respectively. FL1-fluorescent channel 1; FL2-fluorescent channel 2; FITC-fluorescein isothiocyanate; EB-ethidium bromide.
(TIF)

S2 Fig Rabbit antibodies raised against F51-69 (A), F96-111 (B) F270-288 peptides (C), recombinant FliC_Bp_ (D), and prebleed serum (E) recognize crude *B*. *pseudomallei*, recombinant FliC_Bp_ and predicted peptides (A-D). Results are represented by pre-bleed subtracted O.D. (O.D.antisera-O.D.pre-bleed) with error bar. Experiments were performed in duplicate.
(TIF)

S3 Fig *B*. *pseudomallei* protein and peptides neutralize human antibody activity. Crude *B*. *pseudomallei* antigens, FliC_Bp_ peptide F51-69, F96-111, F270-288, F213-231 were coated onto a 96-well polystyrene plate and probed with peptide/protein pre-incubated plasma samples and quantified by Indirect ELISA. Results are represented as Absorbance index (O.D. test-O.D. uncoated / O.D. uncoated). Experiments were performed in duplicate.
(TIF)

S4 Fig Structural comparison of FliC_Bp_ and FliC_Pa_. Secondary structure representation of the superimposed 3D structures of FliC_Bp_ (magenta ribbons) and FliC_Pa_ (PDB entry 4NX9; cyan ribbons). Superimposition of the C-alpha atoms of the two structures was carried out using the C-alpha match server (http://bioinfo3d.cs.tau.ac.il/c_alpha_match/) [68]. This figure was produced using Pymol.
(TIFF)

S5 Fig Comparison of structure-based *in silico* epitope predictions with *in vitro* immunocaptured peptides and synthesized T-cell/B-cell peptides. FliC_Bp_ full-length sequence illustrating epitope residues predicted by MLCE (blue), EDP (green) from the FliC_Bp_ crystal structure, the consensus of three (BepiPred, BCPred and AAC) B-cell sequence-based prediction servers (purple), experimentally mapped peptides (red) and the four synthesized peptides (orange) that represent the consensus of both B-cell and T-cell online sequence-based predictors, and the MLCE-identified peptide. Grey shaded boxes indicate residues shared between more than two or more independent identification methods. The N- and C-terminal residues visible in the electron density of the structure are underlined.
(TIF)

S1 Table Data collection and refinement statistics for FliC_Bp_. A single data collection allowed solving the structure. R_merge_ = ΣI-*(I)* Σ I x 100, where I is the intensity of a reflection and *(I)* is the average intensity; R_free_ was calculated from 5% of randomly selected data for cross-validation; R-factor = ΣF_o_-F_c_/ΣF_o_ x 100. ^a^Values in parentheses refer to the highest resolution shell (1.32–1.3Å).
(DOCX)
