# Supplementary figures and images for: Sequence- and Structure-Based Immunoreactive Epitope Discovery for Burkholderia pseudomallei Flagellin
Source: PLoS Negl Trop Dis. 2015 Jul 29;9(7):e0003917. doi: 10.1371/journal.pntd.0003917 (PMC4519301; doi:10.1371/journal.pntd.0003917)

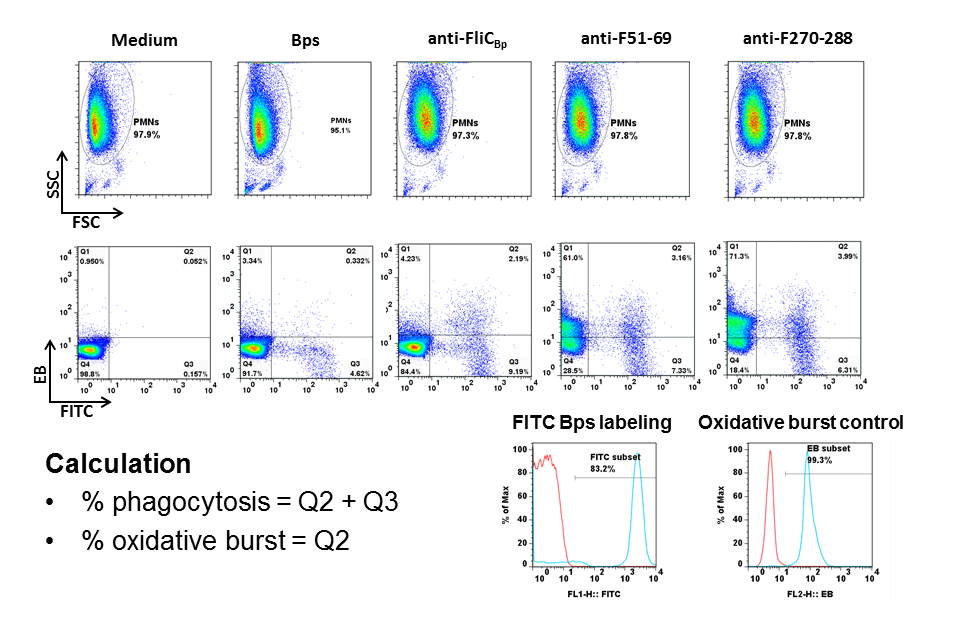

Supplement: S1 Fig — Purified PMN cells were gated on the basis of their FSC/SSC. All gates were identical in all samples within each experiment. The degree of phagocytosis and oxidative burst is shown by fluorescent intensities of FL1-FITC and FL2-EB, respectively. FL1-fluorescent channel 1; FL2-fluorescent channel 2; FITC-fluorescein isothiocyanate; EB-ethidium bromide. (TIF) [file pntd.0003917.s001.tif]

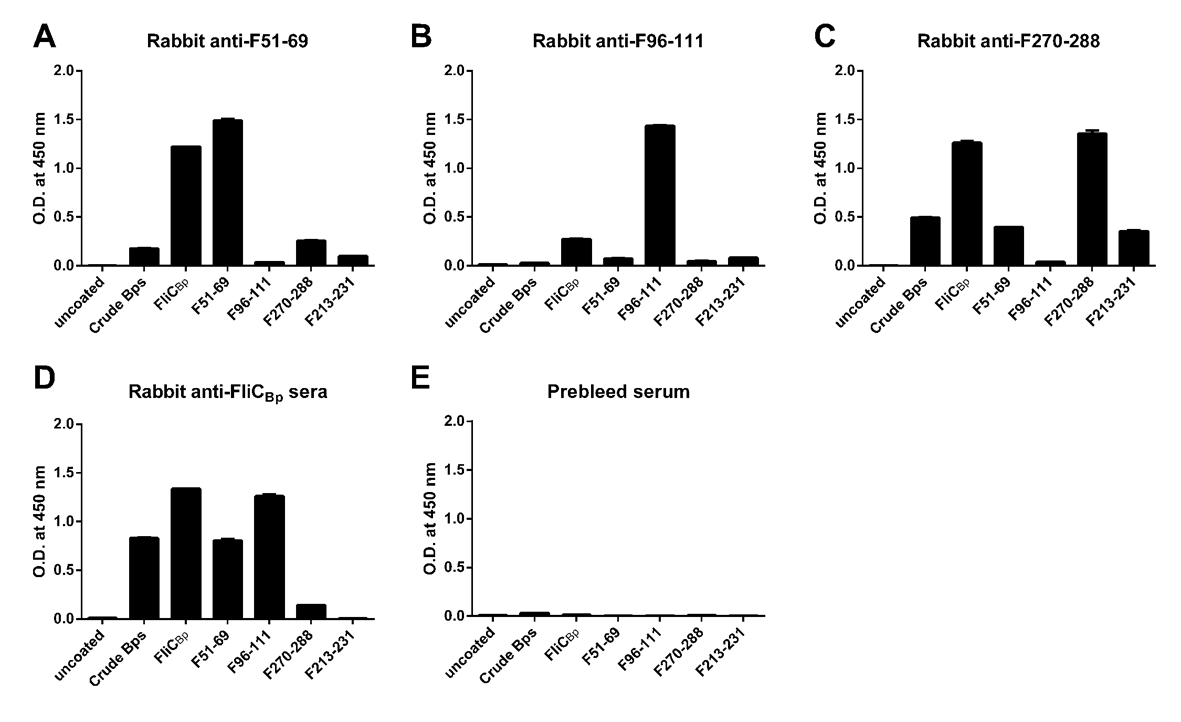

Supplement: S2 Fig — Results are represented by pre-bleed subtracted O.D. (O.D.antisera-O.D.pre-bleed) with error bar. Experiments were performed in duplicate. (TIF) [file pntd.0003917.s002.tif]

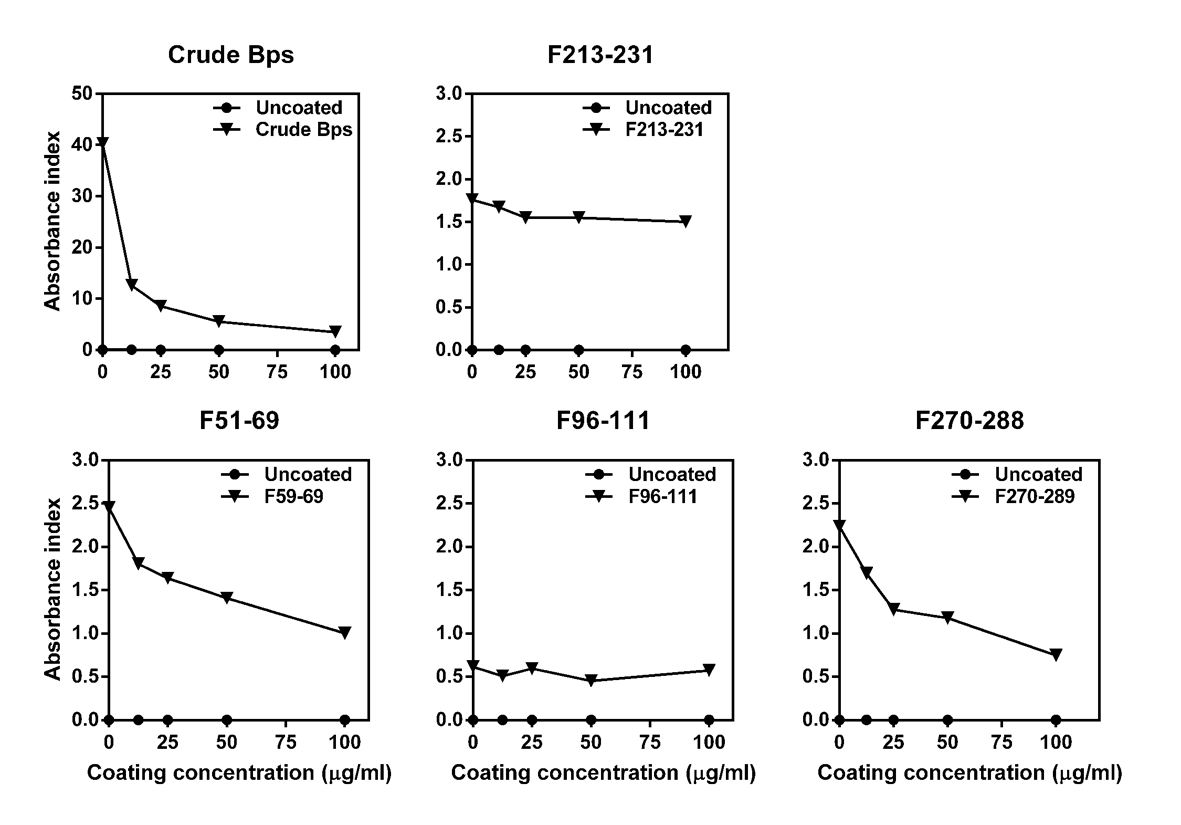

Supplement: S3 Fig — Crude B. pseudomallei antigens, FliCBp peptide F51-69, F96-111, F270-288, F213-231 were coated onto a 96-well polystyrene plate and probed with peptide/protein pre-incubated plasma samples and quantified by Indirect ELISA. Results are represented as Absorbance index (O.D. test-O.D. uncoated / O.D. uncoated). Experiments were performed in duplicate. (TIF) [file pntd.0003917.s003.tif]

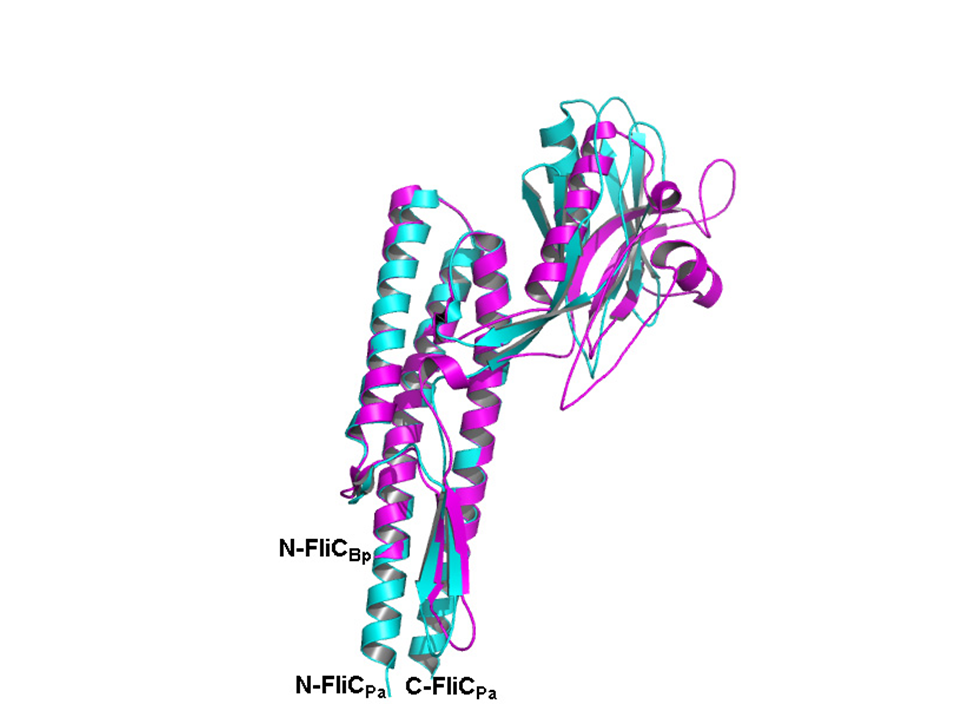

Supplement: S4 Fig — Secondary structure representation of the superimposed 3D structures of FliCBp (magenta ribbons) and FliCPa (PDB entry 4NX9; cyan ribbons). Superimposition of the C-alpha atoms of the two structures was carried out using the C-alpha match server (http://bioinfo3d.cs.tau.ac.il/c_alpha_match/) [68]. This figure was produced using Pymol. (TIFF) [file pntd.0003917.s004.tiff]

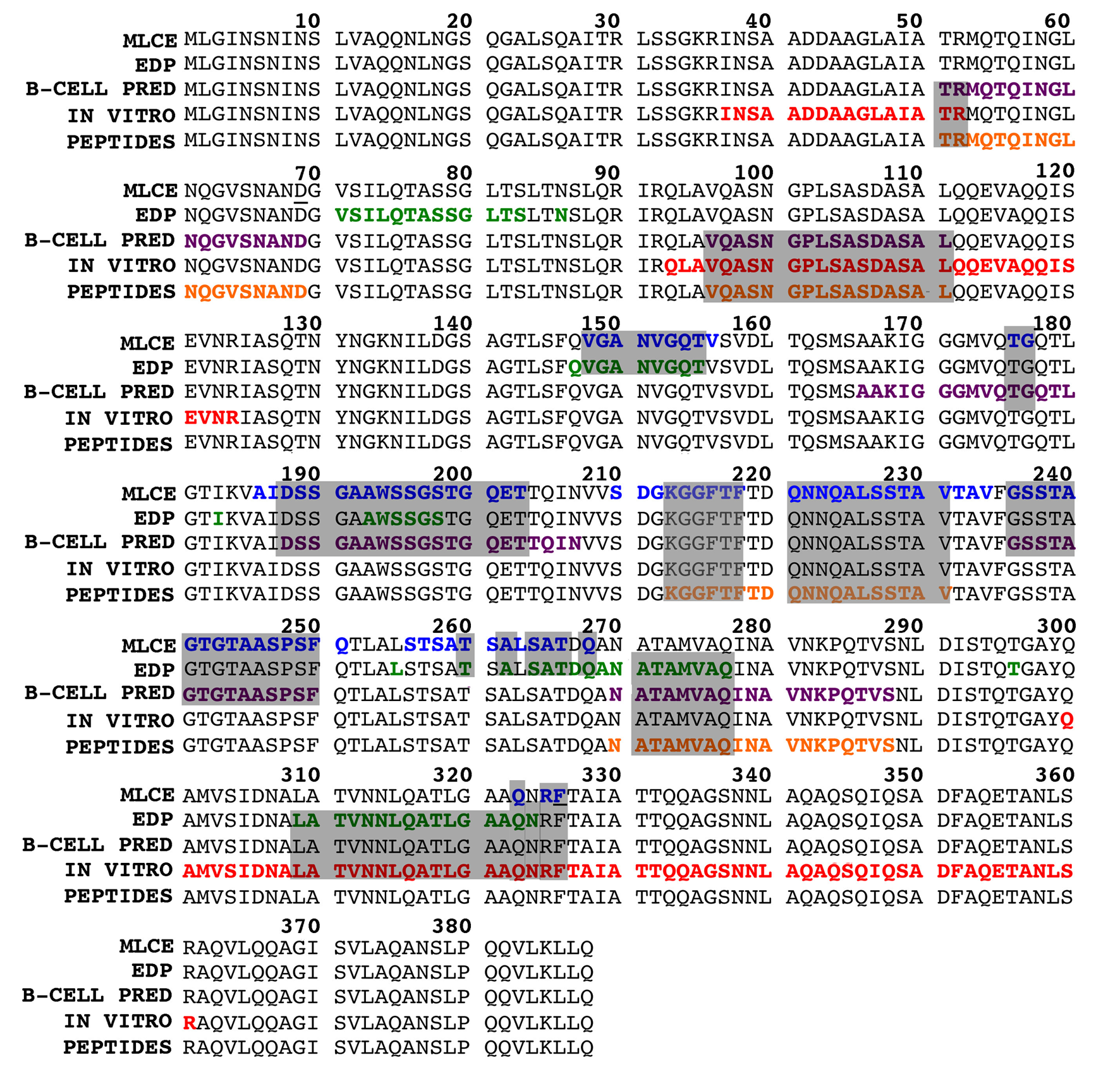

Supplement: S5 Fig — FliCBp full-length sequence illustrating epitope residues predicted by MLCE (blue), EDP (green) from the FliCBp crystal structure, the consensus of three (BepiPred, BCPred and AAC) B-cell sequence-based prediction servers (purple), experimentally mapped peptides (red) and the four synthesized peptides (orange) that represent the consensus of both B-cell and T-cell online sequence-based predictors, and the MLCE-identified peptide. Grey shaded boxes indicate residues shared between more than two or more independent identification methods. The N- and C-terminal residues visible in the electron density of the structure are underlined. (TIF) [file pntd.0003917.s005.tif]
