# Supplementary material for: Sequence- and Structure-Based Immunoreactive Epitope Discovery for Burkholderia pseudomallei Flagellin
Source: PLoS Negl Trop Dis. 2015 Jul 29;9(7):e0003917. doi: 10.1371/journal.pntd.0003917 (PMC4519301; doi:10.1371/journal.pntd.0003917)
Supplement: S1 Table — A single data collection allowed solving the structure. Rmerge = ΣI-(I) Σ I x 100, where I is the intensity of a reflection and (I) is the average intensity; Rfree was calculated from 5% of randomly selected data for cross-validation; R-factor = ΣFo-Fc/ΣFo x 100. aValues in parentheses refer to the highest resolution shell (1.32–1.3Å). (DOCX) [file pntd.0003917.s006.docx]

|  | **FliC_Bp_ (residues 69-326)** |
| --- | --- |
| **Data collection** |  |
| Space group | P2_1_2_1_2_1_ |
| Cell dimensions |  |
| *a*, *b*, *c* (Å) | 23.47 72.78 125.6 |
| α, β, γ (°) | 90 90 90 |
| Resolution (Å) | 24.26-1.3 (1.32-1.3) |
| ^a^*R*_merge_ | 0.052 (0.275) |
| ^a^*I* / σ*I* | 14 (4.8) |
| ^a^Completeness (%) | 99.7 (99.3) |
| ^a^Redundancy | 4.3 (3.9) |
|  |  |
| **Refinement** |  |
| Resolution (Å) | 23.83-1.3 |
| No. reflections | 51380 |
| *R*_gen_ / *R*_free_ | 13.6/16.5 |
| No. atoms |  |
| Protein | 2133 |
| Water | 337 |
| *B*-factors (Å^2^) |  |
| Protein |  |
| Water |  |
| R.m.s. deviations |  |
| Bond lengths (Å) | 0.008 |
| Bond angles (°) | 1.172 |
| Ramachandran Plot (%)  Favoured regions  Allowed regions |  |

**Table S1: Data collection and refinement statistics for FliC_Bp_**
